# Incidence, Trends and Ethnic Differences of Oropharyngeal, Anal and Cervical Cancers: Singapore, 1968-2012

**DOI:** 10.1371/journal.pone.0146185

**Published:** 2015-12-31

**Authors:** Jennifer O. Lam, Wei-Yen Lim, Khuan-Yew Chow, Gypsyamber D’Souza

**Affiliations:** 1 Bloomberg School of Public Health, Johns Hopkins University, Baltimore, MD, United States of America; 2 Saw Swee Hock School of Public Health, National University of Singapore, Singapore, Singapore; 3 National Registry of Diseases Office, Singapore, Singapore; National Health Research Institutes, TAIWAN

## Abstract

In recent decades, several Western countries have reported an increase in oropharyngeal and anal cancers caused by human papillomavirus (HPV). Trends in HPV-associated cancers in Asia have not been as well described. We describe the epidemiology of potentially HPV-related cancers reported to the Singapore Cancer Registry from 1968–2012. Analysis included 998 oropharyngeal squamous cell carcinoma (OPSCC), 183 anal squamous cell carcinoma (ASCC) and 8,019 invasive cervical cancer (ICC) cases. Additionally, 368 anal non-squamous cell carcinoma (ANSCC) and 2,018 non-oropharyngeal head and neck carcinoma (non-OP HNC) cases were included as comparators. Age-standardized incidence rates (ASR) were determined by gender and ethnicity (Chinese, Malay and Indian). Joinpoint regression was used to evaluate annual percentage change (APC) in incidence. OPSCC incidence increased in both genders (men 1993–2012, APC = 1.9%, p<0.001; women 1968–2012, APC = 2.0%, p = 0.01) and was 5 times higher in men than women. In contrast, non-OP HNC incidence declined between 1968–2012 among men (APC = -1.6%, p<0.001) and women (APC = -0.4%, p = 0.06). ASCC and ANSCC were rare (ASR = 0.2 and 0.7 per 100,000 person-years, respectively) and did not change significantly over time except for increasing ANSCCs in men (APC = 2.8%, p<0.001). ICC was the most common HPV-associated cancer (ASR = 19.9 per 100,000 person-years) but declined significantly between 1968–2012 (APC = -2.4%). Incidence of each cancer varied across ethnicities. Similar to trends in Western countries, OPSCC incidence increased in recent years, while non-OP HNC decreased. ICC remains the most common HPV-related cancer in Singapore, but Pap screening programs have led to consistently decreasing incidence.

## Introduction

Human papillomavirus (HPV), a common sexually transmitted infection, causes approximately 4.8% of all cancers worldwide, including nearly 100% of cervical cancers, most (90%) anal cancers, and 35–80% of oropharyngeal cancers.[1–5] Epidemiologic and molecular data indicate that HPV is an increasingly important risk factor for cancer, especially as tobacco-related cancers decline in many countries.[4, 6–8]

The epidemiology of HPV-associated cancers varies geographically and between racial/ethnic groups, likely due to differences in patterns of tobacco use or in sexual behaviors that lead to HPV infection.[1, 4, 8–15] Accumulating evidence from countries in North America and Europe show that the incidence of HPV-associated oropharyngeal squamous cell carcinoma (OPSCC) and anal squamous cell carcinoma (ASCC) has increased over the past 2 to 4 decades, particularly among men.[6, 7, 9, 10, 12–19] Data on the epidemiology of HPV-associated cancers in Asia are more limited, but cancer registry-based studies in Korea and Taiwan found similar increases in HPV-related OPSCC.[20, 21] Trends in other Asian countries, and across Asian ethnic groups, have not been previously studied. Ethnic disparities have been reported in Singapore and other parts of Asia for some infection-associated cancers including cervical,[22, 23] nasopharyngeal[22–24] and liver cancers,[22, 23] but are less clear for oropharyngeal and anal cancers.

To contribute Asian data on this topic, we sought to characterize the epidemiology of potentially HPV-associated cancers in Singapore, focusing on oropharyngeal, anal and cervical cancers. Singapore is a high-income, multicultural city-state of 4 million residents in Southeast Asia with 3 primary ethnicities—Chinese (74.2%), Malay (13.3%) and Indian (9.2%).[25] We evaluated potentially HPV-associated cancers in Singapore over 4 decades to: 1) determine incidence of cervical, oropharyngeal and anal cancers, by type, gender and ethnicity, and 2) characterize temporal trends in these cancers.

## Methods

### Case inclusion and classification

We restricted our analysis to confirmed incident cancers at anatomic sites where HPV is known to be a primary cause, including oropharyngeal squamous cell carcinoma (OPSCC), anal squamous cell carcinoma (ASCC) and invasive cervical cancer (ICC). We included as comparators, non-oropharyngeal head and neck squamous cell carcinoma (non-OP HNC), which is primarily tobacco-related, and invasive anal non-squamous cell carcinoma (ANSCC).

Cancer sites were defined according to International Classification of Diseases codes for oncology (ICD-O-3). Tumor HPV status was not available, so tumor site was used as a proxy to classify cases as “HPV-related” and “HPV-unrelated”, similar to previous research.[6, 8, 9, 14, 16–18, 20, 21, 26] OPSCC sites included the oropharynx (C10.0-C10.4, C10.8-C10.9), tonsil (C02.4, C09.0-C09.1, C09.8-C09.9), base of tongue (C01.9), soft palate and uvula (C05.1-C05.2), and Waldeyer’s ring (C14.2). Non-OP HNC sites included other parts of the tongue (C02.0-C02.3, C02.8-C02.9), mouth (C04.0-C04.1, C04.8-C04.9; C06.0-C06.2, C06.8-C06.9), gum (C03.0-C03.1, C03.9), and hard palate (C05.0, C05.8-C05.9). OPSCC and non-OP HNC analyses were restricted to cancers with squamous cell histologies (ICD-O-3 codes: 8050 to 8076, 8078, 8083, 8084, 8094). ICC included endocervix (C53.0), exocervix (C53.1), and cervix uteri (C53.8, C53.9). All histologic types of ICC were included as nearly all ICC is due to HPV infection.[5] Invasive anal cancers (C21.0-C21.8) were subdivided into squamous and non-squamous histology.

### Data sources

The numbers of newly diagnosed cancer cases reported between 1968–2012 were obtained from the Singapore Cancer Registry, a population-based registry covering all Singapore residents. The Ministry of Health Singapore enacted the National Registry of Diseases Act in 2007 to ensure comprehensive notifications of cancer cases (local and foreign residents) by healthcare institutions in Singapore. The Singapore Cancer Registry includes 1.09% death certificate only cases and 91.8% morphologically verified cases (unpublished information). Case counts were obtained in aggregate form, by 5-year calendar period (i.e. 1968–72, 1973–77…2008–2012) and age groups (i.e. 20–24, 25–29…65–69, ≥70) (S1 Data). Data were further subdivided by gender and ethnicity. Cancer registry information was based on data from medical professionals, pathology records and hospital records.[23] Population denominators for incidence rates were derived from mid-year population estimates from the Singapore Department of Statistics for each year. Our analysis used aggregated, de-identified patient data only. Permission was obtained from the Singapore National Diseases Registry Office and was approved as exempt from IRB review by the National University of Singapore IRB.

### Statistical analyses

Crude incidence rates for each 5-year period were calculated for each cancer type, overall and by gender and ethnicity. Age-standardized incidence rates (ASR) per 100,000 person-years were calculated using the direct method[27] and based on the WHO world standard population.[28] Incidence rate ratios (IRRs) compared men and women overall and for each cancer type. Stata 12 software was used.[29]

Temporal trends in ASR (5-year periods) for each cancer were characterized using the Joinpoint Regression Program, version 4.1.1.[30] This method uses least squares regression to fit line segments to the natural log of the ASR, joined at discrete points (midpoint of 5-year periods) identified by the software to represent statistically significant changes in direction of trend.[30] The average annual percentage change (APC) in ASR was calculated and considered significant at p≤0.05. Temporal trends were explored by gender for all cancer types. Temporal trends in ICC were also explored when stratified by Chinese, Malay and Indian ethnicities. The “Other ethnicity” category was excluded from ethnicity-stratified analyses because the number of cancers was too few for reliable results, but was included in overall and gender-stratified analyses. Due to low numbers, ethnicity-stratified Joinpoint results are not reported for OPSCC, non-OP HNC and anal cancers.

## Results

Between 1968–2012, 9,200 potentially HPV-associated cancers were diagnosed in Singapore including 998 OPSCC, 183 ASCC and 8,019 ICC. There were 2,018 non-OPC HNC and 368 ANSCC diagnosed during the same period (Table 1). The incidence of each cancer increased with age, and the median age at diagnosis for OPSCC, non-OP HNC, ASCC, ANSCC and ICC was 62, 61, 66, 66 and 53 years, respectively.

**Table 1:** Trends in crude cancer incidence over time, by type and gender, from 1968 to 2012 in Singapore^a^.

			Oropharyngeal SCC		Non-oropharyngeal HNC		Anal SCC		Anal non-SCC		ICC^b^	
Gender	Year of diagnosis	Person-years	n	Incidence (per 100,000)	n	Incidence (per 100,000)	n	Incidence (per 100,000)	n	Incidence (per 100,000)	n	Incidence (per 100,000)
**Men**	**1968–1972**	2,608,200	59	2.26	93	3.57	4	0.15	5	0.19	NA	NA
	**1973–1977**	3,111,100	59	1.90	110	3.54	9	0.29	8	0.26	NA	NA
	**1978–1982**	3,576,850	55	1.54	123	3.44	4	0.11	7	0.20	NA	NA
	**1983–1987**	4,108,400	64	1.56	145	3.53	7	0.17	17	0.41	NA	NA
	**1988–1992**	4,700,300	74	1.57	135	2.87	8	0.17	23	0.49	NA	NA
	**1993–1997**	5,276,530	76	1.44	156	2.96	6	0.11	18	0.34	NA	NA
	**1998–2002**	5,784,960	119	2.06	194	3.35	9	0.16	25	0.43	NA	NA
	**2003–2007**	6,216,165	139	2.24	181	2.91	11	0.18	49	0.79	NA	NA
	**2008–2012**	6,871,900	183	2.66	213	3.10	19	0.28	69	1.00	NA	NA
	**TOTAL**	**42,254,405**	**828**	**1.96**	**1,350**	**3.19**	**77**	**0.18**	**221**	**0.52**	**NA**	**NA**
**Women**	**1968–1972**	2,477,300	4	0.16	39	1.57	4	0.16	4	0.16	603	24.34
	**1973–1977**	3,020,100	4	0.13	34	1.13	5	0.17	7	0.23	676	22.38
	**1978–1982**	3,519,250	15	0.43	53	1.51	12	0.34	2	0.06	751	21.34
	**1983–1987**	4,079,100	10	0.25	56	1.37	10	0.25	11	0.27	896	21.97
	**1988–1992**	4,682,260	14	0.30	68	1.45	9	0.19	11	0.23	998	21.31
	**1993–1997**	5,349,900	15	0.28	84	1.57	6	0.11	25	0.47	1,130	21.12
	**1998–2002**	5,960,376	23	0.39	102	1.71	25	0.42	23	0.39	1,038	17.42
	**2003–2007**	6,480,899	33	0.51	100	1.54	15	0.23	29	0.45	1,014	15.65
	**2008–2012**	7,200,600	52	0.72	132	1.83	20	0.28	35	0.49	913	12.68
	**TOTAL**	**42,769,785**	**170**	**0.40**	**668**	**1.56**	**106**	**0.25**	**147**	**0.34**	**8,019**	**18.75**
**All**	**All**	**85,024,190**	**998**	**1.17**	**2,018**	**2.37**	**183**	**0.22**	**368**	**0.43**	**8,019**	**18.75**

^a^ Non-age-standardized incidence per 100,000 person-years

^b^ Only women are included in the person-years denominator when calculating incidence of ICC

Abbreviations: SCC = squamous cell carcinoma; HNC = head and neck squamous cell carcinoma; ICC = invasive cervical cancer; n = number of cases

Gender disparities in age-standardized incidence rates were observed for most cancer types. OPSCC, non-OPC HNC and ANSCC occurred significantly more frequently in men than women, while ASCC incidence was similar in men and women (Table 2). ICC accounted for 87% of all HPV-associated cancers (ASR = 19.9 per 100,000 person-years). The incidence of OPSCC (ASR = 1.4 per 100,000 person-years) and ASCC (ASR = 0.3 per 100,000 person-years) were lower.

**Table 2:** Age-standardized incidence rates (ASR) per 100,000 person years for each cancer, overall and by gender, from 1968 to 2012 in Singapore.

	Total		Men		Women		Incidence rate ratio
							(95% CI)
Cancer type	No. of cases	ASR^a^	No. of cases	ASR^a^	No. of cases	ASR^a^	Men: Women
**Oropharyngeal SCC**	998	1.38	828	2.44	170	0.44	5.54 (4.69–6.54)
**Non-oropharyngeal HNC**	2,018	2.77	1,350	3.91	668	1.74	2.25 (2.05–2.47)
**Anal SCC**	183	0.26	77	0.23	106	0.29	0.80 (0.59–1.07)
**Anal non-SCC**	368	0.52	221	0.67	147	0.40	1.69 (1.37–2.09)
**Invasive cervical cancer**	8,019	19.92	NA	NA	8,019	19.92	NA
**Oropharyngeal & Anal SCC**	**1,181**	**1.64**	**905**	**2.67**	**276**	**0.73**	**3.68 (3.21–4.21)**
**HPV-associated cancers** ^b^	**9,200**	**11.83**	**905**	**2.67**	**8,295**	**20.65**	**0.13 (0.12–0.14)**

^**a**^ Age-standardized incidence rates per 100,000 person-years; Age-standardization was done using the direct method and based on the WHO world standard population

^b^ HPV-associated cancers include oropharyngeal SCC, anal SCC and invasive cervical cancer.

Abbreviations: CI = confidence interval; non-OP HNC = non-oropharyngeal head and neck squamous cell carcinoma; SCC = squamous cell carcinoma

### Incidence trends

**Fig 1:**
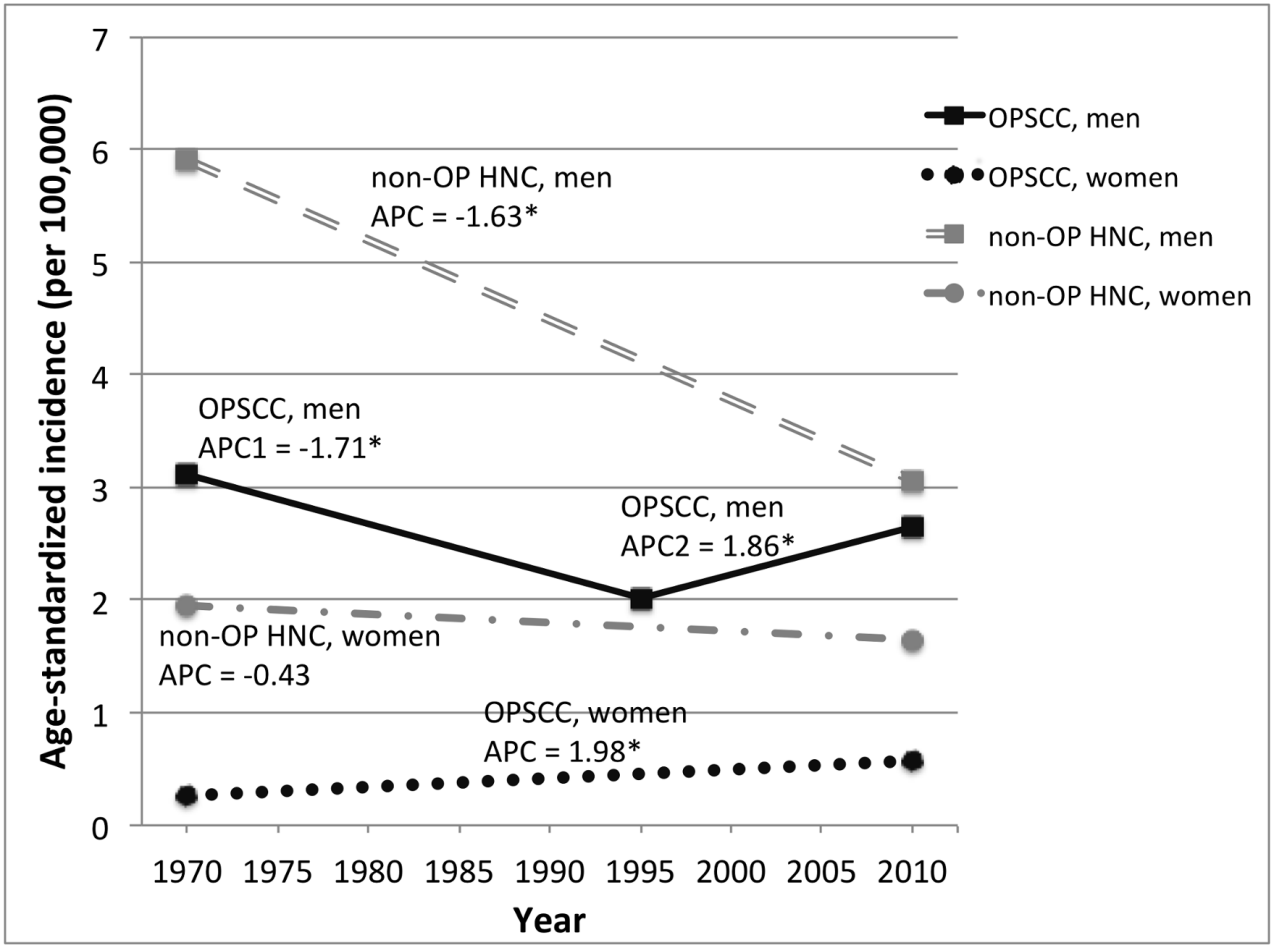
Incidence of oropharyngeal and non-oropharyngeal head and neck squamous cell carcinomas in Singapore 1968–2012, by gender. Incidence trends are based on incidence rates for 5-year time periods that were age-adjusted to the WHO standard population. Annual percent change (APC) was calculated using Joinpoint regression analysis. APC = annual percent change. An asterisk (*) indicates an APC value that is statistically significant at p≤0.05. Abbreviations: OPSCC = oropharyngeal squamous cell carcinoma; non-OP HNC = non-oropharyngeal head and neck squamous cell carcinoma

**Fig 2:**
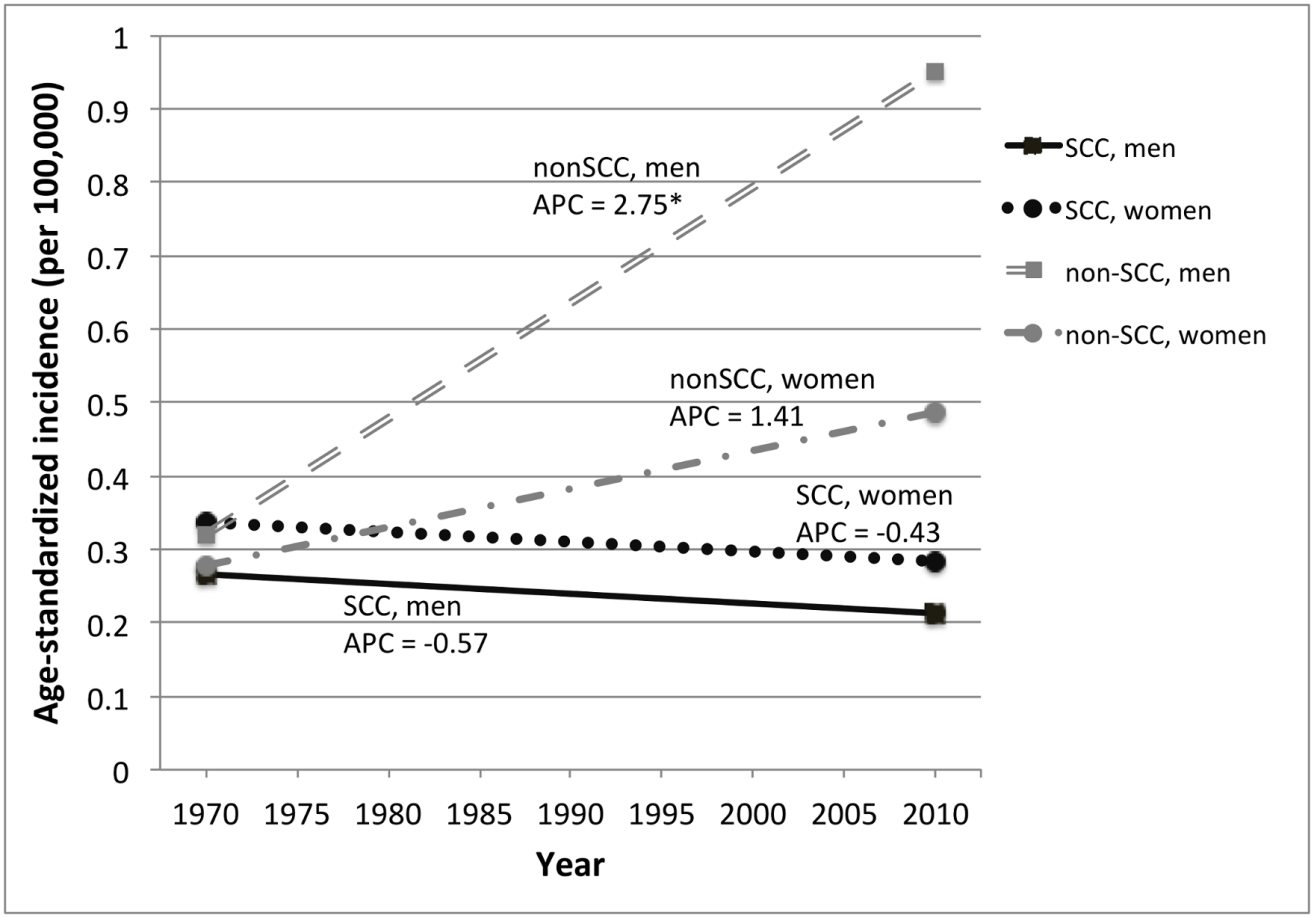
Incidence of invasive anal cancer in Singapore, 1968–2012, by gender and histology. Incidence trends are based on incidence rates for 5-year time periods that were age-adjusted to the WHO standard population. Annual percent change (APC) was calculated using Joinpoint regression analysis. APC = annual percent change. An asterisk (*) indicates an APC value that is statistically significant at p≤0.05. Abbreviations: SCC = squamous cell carcinoma, non-SCC = non-squamous cell carcinoma

**Fig 3:**
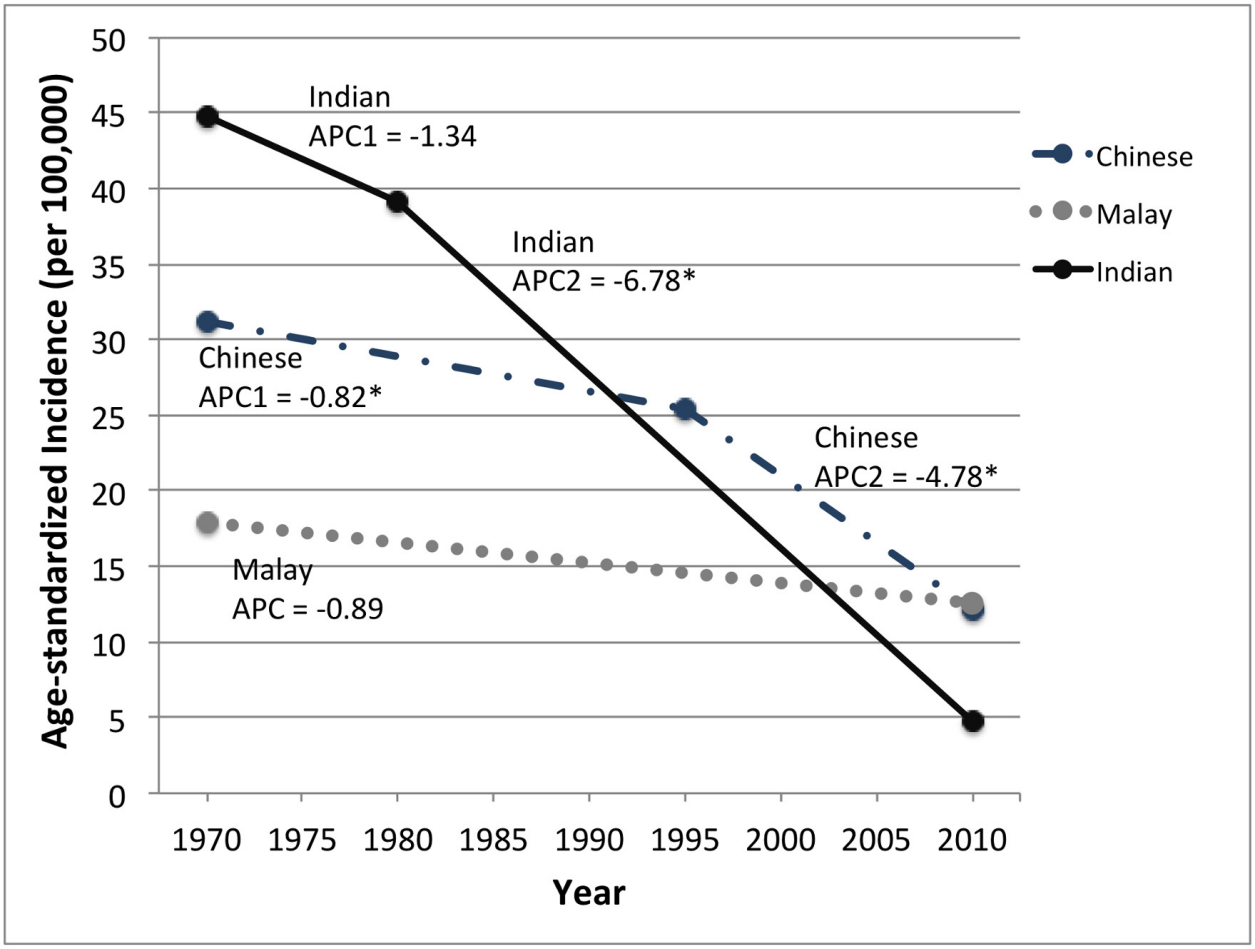
Incidence of invasive cervical cancer in Singapore, 1968–2012, by ethnicity. Incidence trends are based on incidence rates for 5-year time periods that were age-adjusted to the WHO standard population. Annual percent change (APC) was calculated using Joinpoint regression analysis. APC = annual percent change. An asterisk (*) indicates an APC value that is statistically significant at p≤0.05.

Trends in OPSCC and non-OP HNC (Fig 1), ASCC and ANSCC (Fig 2), and ICC (Fig 3) between 1968–2012 were explored. In the last 20 years (1993–2012), OPSCC incidence increased steadily in men (APC = 1.9%, p<0.001) and women (APC = 2.0%, p = 0.01) (Fig 1). In contrast, non-OP HNC incidence decreased in men (APC = -1.6, p<0.001) and women (APC = -0.4, p = 0.06) during this time period. In previous time periods (1968–1992), the incidence of both OPSCC and non-OP HNC decreased in men, but only decreased for non-OP HNC in women (Fig 1).

Analyses of anal carcinomas by histologic subtype suggested different trends over time in ASCC and ANSCC. ASCC incidence appeared to decrease from 1968 to 2012 in both genders. In contrast, ANSCC incidence appeared to increase during this same period (Fig 2). The increase in ANSCC incidence in men was significant (APC = 2.8%, p<0.001), but other observed ASCC and ANSCC trends were not statistically significant.

ICC incidence declined consistently across all ethnicities, most notably in Indian women, who experienced an average -6.78% (p<0.001) decrease in ASR per year between 1978–2012 and an overall 9.5-fold decrease in incidence from 44.8 to 4.7 per 100,000 person-years between 1968–2012 (Fig 3). ICC incidence also decreased in Chinese women, primarily from 1993–2012 (APC = -4.8%, p<0.001). In the most recent time period (2008–2012), the overall incidence of ICC in Singapore (ASR = 11.6 per 100,000 person-years) remained lower than the global estimated burden (ASR = 15.2 per 100,000 person-years) but slightly higher than the average rate in more developed countries (ASR = 9.0 per 100,000 person-years).[31]

### Ethnic differences

Ethnic differences in the incidence of potentially HPV-associated cancers (OPC, ASCC and ICC) were observed. Chinese women had higher risk of HPV-associated cancer overall (ASR = 22.0 per 100,000 person-years) compared to Malay (ASR = 14.7, p<0.001) or Indian (ASR = 14.9, p<0.001) women, primarily due to higher ICC incidence in Chinese women (Table 3). In contrast, Indian men had higher risk of HPV-associated cancer (ASR = 4.0 per 100,000 person-years) compared to Chinese (ASR = 2.7, p<0.001) or Malay (ASR = 1.1, p<0.001) men, primarily due to high OPSCC incidence among Indian men (Table 3; S1 Table). When considering only the most recent time period (2008–2012), these same ethnic variations remain.

**Table 3:** Age-standardized incidence rates (ASR) per 100,000 person years for each cancer, by gender and ethnicity, from 1968 to 2012 in Singapore.

	Men						Women					
	Chinese		Malay		Indian		Chinese		Malay		Indian	
Cancer type	No. of cases	ASR^a^	No. of cases	ASR^a^	No. of cases	ASR^a^	No. of cases	ASR^a^	No. of cases	ASR^a^	No. of cases	ASR^a^
**Oropharyngeal SCC**	671	2.53	26	0.72	115	3.64	140	0.44	11	0.31	17	0.96
**Non-oropharyngeal HNC**	963	3.56	78	2.03	283	9.12	453	1.42	67	1.67	142	7.51
**Anal SCC**	48	0.19	15	0.19	13	0.39	92	0.29	15	0.18	5	0.31
**Anal non-SCC**	181	0.70	20	0.55	17	0.57	128	0.41	6	0.41	4	0.20
**Invasive cervical cancer**	NA	NA	NA	NA	NA	NA	6,969	21.29	645	14.17	308	13.67
**Oropharyngeal & Anal SCC**	**719**	**2.72**	**41**	**1.11**	**128**	**4.03**	**232**	**0.73**	**26**	**0.49**	**22**	**1.27**
**HPV-associated cancers** ^b^	**719**	**2.72**	**41**	**1.11**	**128**	**4.03**	**7,201**	**22.03**	**671**	**14.67**	**330**	**14.93**

^**a**^ Age-standardized incidence rates per 100,000 person-years; Age-standardization was done using the direct method and based on the WHO world standard population

^b^ HPV-associated cancers include oropharyngeal SCC, anal SCC and invasive cervical cancer.

Abbreviations: non-OP HNC = non-oropharyngeal head and neck squamous cell carcinoma; SCC = squamous cell carcinoma

## Discussion

This study suggests that there are gender and ethnic differences in the incidence and temporal trends of potentially HPV-associated cancers in Singapore. Incidence of HPV-associated cancer overall was higher in women than men, due to the burden of cervical cancer among women, but OPSCC incidence was significantly higher in men than women. Ethnic differences in incidence of HPV-associated cancer were observed, with higher rates overall among Indian men and among Chinese women than other ethnicities. Over the 45 years studied, ICC and non-OP HNC incidence decreased significantly in Singapore, but OPSCC rates increased in both men and women in recent years. This research suggests the distribution of HPV-associated cancers across population subgroups has changed over the past few decades, possibly mirroring changes in tobacco and sexual risk factors.

Similar to trends reported in other countries of comparable socioeconomic status, the incidence of OPSCC in Singapore is rising.[4, 6–9, 11, 14–21] However, unlike some other countries where this increase was only observed in men,[4, 7, 32] OPSCC incidence in Singapore appeared to increase for both genders. Recent research suggests increases in OPSCC are largely explained by HPV and are likely driven by changing sexual practices.[4, 8, 15, 33] In the current study, tumor HPV status was not available, so we do not know what proportion of the OPSCC cases included in our analysis was HPV-positive and how this proportion differed by gender and ethnicity, or changed over time. Given that the observed patterns in OPSCC incidence differed in men and women, it is possible that changing sexual practices may not fully explain trends.

The recent increasing trend in OPSCC contrasts with consistently decreasing rates of non-OP HNC, which are more strongly tied to tobacco usage. Decreasing OPSCC incidence in men in the 1970s and 1980s is likely related to declining tobacco use, including decreasing popularity of traditional smoking methods such as hand-rolled cigarettes (“ang hoon”). Smoking prevalence in Singapore has been declining in men since the 1970s and has remained low (<5%) in women,[34, 35] consistent with observed decreases in non-OP HNC in this study and decreasing lung cancer rates reported elsewhere.[23, 35] The overall incidence of non-OP HNC in Singapore is substantially lower than that in North America and Europe,[11, 36] consistent with the lower smoking rate in Singapore (~13%).[34] Tobacco use in Singapore is amongst the lowest in developed countries, largely due to the success of anti-tobacco campaigns, legislation on tobacco taxation and prohibition of smoking in public places.[34] Given the low smoking prevalence, the epidemiology of non-OPC in Singapore may reflect the HNC profile we will see in other settings as tobacco cessation efforts continue.

Overall, anal cancer is an uncommon malignancy in Singapore. The observed ASCC and ANSCC rates were 2- to 4-fold lower in Singapore than in Western countries (typically 1–2 cases per 100,000 in the general population), probably reflecting differences in sexual habits in the Singapore population.[12] Unlike some other countries, ASCC and ANSCC in Singapore are more common among men than women, although numbers for both genders are low.[37, 38] The observed increase in incidence of ANSCC is similar to the increasing incidence of colorectal cancer, the most common cancer among men in Singapore, during roughly this same time period.[39] If ANSCC and colorectal cancer have similar risk factors, this could explain the sharp increase in ANSCC observed during the study period. The strongly divergent trends in ASCC and ANSCC over time suggest etiologic differences and the importance of distinguishing anal cancers by histologic type in future reports of cancers at HPV-related subsites.

Despite substantial decreases in the incidence of ICC in Singapore over the past 4 decades, ICC remains the most common HPV-associated cancer in Singapore. The incidence of ICC in Singapore remains high compared to Western countries of similar economic status.[31, 40] However, consistent with previous studies,[23, 40] we observed an encouraging decline in ICC rates during the time period of our study. This is largely attributed to opportunistic Pap screening which has been available since 1964, and a national cervical cancer screening program implemented in 2004, both of which have contributed to early detection and treatment of cervical pre-cancers.[40–42]

The national ICC screening program, which targets sexually active women starting at age 25, has successfully expanded coverage with comparable reach for Chinese, Malay and Indian ethnicities for first screens.[40, 43] Health surveys conducted in 2008 and 2010 found that Malay women had a higher rate of loss to re-screen, followed by Indian and then Chinese women, which may explain why ICC rates have decreased the least in Malay women.[41, 43] However, it is unclear why Chinese women remain at higher risk of ICC, as compared to Malay and Indian women in Singapore. In the future, increasing Pap screening coverage and timely re-screens may reduce ICC rates further.[41] HPV vaccines are licensed for use in Singapore, but must be covered by out-of-pocket expenses, or Medisave, a compulsory health savings scheme where individuals put aside part of their income to pay future medical expenses for themselves or dependents.[40, 44] Implementation of a national HPV vaccination program or provision of subsidies for low-income individuals could also contribute to future declines in incidence of ICC, and other HPV-associated cancers.

Although women experience the greater HPV-associated cancer burden in Singapore, our results suggest that men may also benefit from prevention efforts targeting these cancers as they bear a greater risk of OPSCC. Compared to other cancers in Singapore,[23] OPSCC is relatively rare, and rates in Singapore are lower than in other economically developed countries.[7, 11] However, men are disproportionately affected and rates are increasing. Should HPV prevention efforts be scaled up in Singapore, our study provides baseline data on the incidence of potentially HPV-associated cancers during time periods when HPV vaccination coverage is minimal.

Although the cancers in our analysis are at HPV-related sites, HPV may be just one of the factors contributing to observed cancer epidemiology. The heterogeneity in patterns of HPV-associated cancer that we observed across ethnic groups could reflect the effects of sociocultural practices, genetics, environmental exposures or an interaction between etiologic factors. Since the 1960s, Singapore has undergone rapid economic development and has become increasingly ‘westernized’ in its transition from a developing country to a high-income country and commercial hub in Southeast Asia. This has been accompanied by changes in diet, lifestyle and customs that have been cited as contributing factors to the increasing burden of chronic infections and cancer.[39, 40, 44–46]

Differences between ethnic groups in lifestyle factors such as tobacco use and sexual norms may contribute to observed differences in HPV-related cancer incidence. For instance, Indians are known to have a high prevalence of tobacco and betel quid use, practices which increase risk of head and neck cancers.[47–50] The higher incidence of OPSCC observed in Indian Singaporean men and women could be due to early life exposure to betel quid in their country of origin or continuation of betel use habits after immigration to Singapore. Lower incidence of non-OP HNC among Malays is surprising given that their smoking rates are higher than the general Singaporean population (18.6% to 30.8% between 1979–2010),[35] but Malays also have low lung cancer incidence suggesting that factors other than smoking exposure may account for differences in cancer incidence.[35, 49] Overall, Malays have the lowest HPV-associated cancer incidence, compared to Chinese and Indian ethnic groups. This may be due to lower risk of HPV infection, or possibly other lifestyle factors that may be protective of cancers. Most Malays are Muslim, and they may adhere to a more traditional lifestyle (i.e. diet, sexual behaviors) despite Singapore’s modern environment.[51] It is also possible that ethnicity does not fully encompass behavioral, sociocultural and genetic differences and that the broad ethnic categorizations used may obscure relevant within-group differences in practices or behaviors.

Limitations of this study include low numbers of OPSCC and ASCC, and lack of data on tumor HPV status and behavioral risk factors, including smoking and betel quid use. Additionally, some cases had insufficient information to identify a precise tumor site and were classified as ICD-O-3 NOS (“not otherwise specified” tumor site) in the Singapore Cancer Registry; thus, misclassification of these cases is another potential limitation. Strengths of this study include the use of high-quality cancer registry data that is representative of the Singaporean resident population, the long time-interval (>40 years) included in the analysis, and the Asian ethnic variation explored.

## Conclusions

Our study provides a snapshot of the current burden and recent trends of oropharyngeal, anal and cervical cancers in Singapore, a multi-ethnic setting where HPV vaccination is not yet widespread. Although HPV-associated cancer prevention in Singapore has primarily focused on cervical cancer, our study shows for the first time that there is also a burden of potentially HPV-related oropharyngeal cancers in men, and that the incidence is rising. Furthermore, our study illustrates substantial differences in burden of these cancers by Asian ethnicities, underscoring the need to understand differences in risk factors across population subgroups in Singapore’s diverse setting. With progressively increasing industrialization and population growth, the epidemiology of these cancers in Singapore may reflect the cancer profile we will see in other settings as tobacco cessation efforts continue. Understanding the changing epidemiology of HPV-associated cancers is important for cancer prevention and provides a picture of cancer risk in a population with low tobacco use.

## Supporting Information

S1 Table Trends in crude and age-standardized oropharyngeal squamous cell carcinoma (OPSCC) incidence over time, by ethnicity and gender, from 1968 to 2012 in Singapore. ^a^ cIR = Crude (non-age standardized) incidence per 100,000 person-years. ^b^ ASR = Age-standardized incidence per 100,000 person-years.
(DOCX)

S1 Data Aggregate case counts of oropharyngeal squamous cell carcinoma (OPSCC), non-oropharyngeal head and neck carcinoma (non-OP HNC), invasive cervical cancer (ICC), anal squamous cell carcinoma (ASCC) and anal non-squamous cell carcinoma (ANSCC), by sex, ethnicity, age group and time period, from 1968 to 2012 in Singapore. (XLSX)
