# Supplementary material for: Incidence, Trends and Ethnic Differences of Oropharyngeal, Anal and Cervical Cancers: Singapore, 1968-2012
Source: PLoS One. 2015 Dec 31;10(12):e0146185. doi: 10.1371/journal.pone.0146185 (PMC4705110; doi:10.1371/journal.pone.0146185)
Supplement: S1 Table — a cIR = Crude (non-age standardized) incidence per 100,000 person-years. b ASR = Age-standardized incidence per 100,000 person-years. (DOCX) [file pone.0146185.s001.docx]

**S1 Table. Trends in crude and age-standardized oropharyngeal squamous cell carcinoma (OPSCC) incidence over time, by ethnicity and gender, from 1968 to 2012 in Singapore.**

|  | | **Chinese** | | | | **Malay** | | | | **Indian** | | | |
| --- | --- | --- | --- | --- | --- | --- | --- | --- | --- | --- | --- | --- | --- |
| **Gender** | **Year of diagnosis** | **Person-years** | **n** | **cIR^a^** | **ASR^b^** | **Person-years** | **n** | **cIR^a^** | **ASR^b^** | **Person-years** | **n** | **cIR^a^** | **ASR^b^** |
| **Men** | **1968-1972** | 1,946,200 | 39 | 2.00 | 2.82 | 339,300 | 3 | 0.88 | 1.77 | 265,400 | 16 | 6.03 | 8.54 |
|  | **1973-1977** | 2,367,600 | 44 | 1.86 | 2.78 | 406,100 | 3 | 0.74 | 2.16 | 265,800 | 11 | 4.14 | 5.27 |
|  | **1978-1982** | 2,775,000 | 40 | 1.44 | 2.27 | 469,900 | 2 | 0.43 | 0.67 | 276,580 | 13 | 4.70 | 6.70 |
|  | **1983-1987** | 3,210,000 | 52 | 1.62 | 2.47 | 542,500 | 1 | 0.18 | 0.26 | 315,600 | 8 | 2.53 | 2.15 |
|  | **1988-1992** | 3,676,900 | 56 | 1.52 | 2.17 | 611,800 | 6 | 0.98 | 1.86 | 366,300 | 12 | 3.28 | 3.73 |
|  | **1993-1997** | 4,152,600 | 63 | 1.52 | 1.97 | 661,600 | 2 | 0.30 | 0.49 | 408,600 | 10 | 2.45 | 2.52 |
|  | **1998-2002** | 4,544,901 | 101 | 2.22 | 2.71 | 700,597 | 5 | 0.71 | 1.00 | 467,146 | 11 | 2.35 | 2.73 |
|  | **2003-2007** | 4,820,243 | 120 | 2.49 | 2.64 | 756,117 | 2 | 0.26 | 0.19 | 522,110 | 14 | 2.68 | 3.39 |
|  | **2008-2012** | 5,195,500 | 156 | 3.00 | 2.75 | 827,400 | 2 | 0.24 | 0.23 | 642,800 | 20 | 3.11 | 3.81 |
|  | **TOTAL** | **32,688,944** | **671** | **2.05** | **2.53** | **5,315,314** | **26** | **0.49** | **0.72** | **3,530,336** | **115** | **3.26** | **3.64** |
| **Women** | **1968-1972** | 1,992,400 | 3 | 0.15 | 0.18 | 314,800 | 0 | 0.00 | 0.00 | 117,200 | 1 | 0.85 | 5.38 |
|  | **1973-1977** | 2,421,200 | 4 | 0.17 | 0.19 | 383,500 | 0 | 0.00 | 0.00 | 151,800 | 0 | 0.00 | 0.00 |
|  | **1978-1982** | 2,826,600 | 11 | 0.39 | 0.48 | 451,100 | 1 | 0.22 | 0.28 | 188,700 | 3 | 1.59 | 3.00 |
|  | **1983-1987** | 3,262,400 | 9 | 0.28 | 0.30 | 529,600 | 1 | 0.19 | 0.25 | 242,500 | 0 | 0.00 | 0.00 |
|  | **1988-1992** | 3,733,100 | 11 | 0.29 | 0.34 | 598,300 | 1 | 0.17 | 0.40 | 298,170 | 2 | 0.67 | 1.65 |
|  | **1993-1997** | 4,266,700 | 8 | 0.19 | 0.21 | 660,200 | 3 | 0.45 | 0.69 | 354,800 | 4 | 1.13 | 1.59 |
|  | **1998-2002** | 4,736,120 | 18 | 0.38 | 0.39 | 713,753 | 1 | 0.14 | 0.12 | 422,463 | 2 | 0.47 | 0.62 |
|  | **2003-2007** | 5,080,665 | 29 | 0.57 | 0.51 | 781,445 | 2 | 0.26 | 0.34 | 487,817 | 2 | 0.41 | 0.65 |
|  | **2008-2012** | 5,529,500 | 47 | 0.85 | 0.71 | 862,300 | 2 | 0.23 | 0.27 | 582,400 | 3 | 0.52 | 0.66 |
|  | **TOTAL** | **33,848,685** | **140** | **0.41** | **0.44** | **5,294,998** | **11** | **0.21** | **0.31** | **2,845,850** | **17** | **0.60** | **0.96** |

^a^ cIR = Crude (non-age standardized) incidence per 100,000 person-years

^b^ ASR = Age-standardized incidence per 100,000 person-years
